# Soybean (Glycine max L Merr) host-plant defenses and resistance to the two-spotted spider mite (Tetranychus urticae Koch)

**DOI:** 10.1371/journal.pone.0258198

**Published:** 2021-10-07

**Authors:** Ian M. Scott, Tim McDowell, Justin B. Renaud, Sophie W. Krolikowski, Ling Chen, Sangeeta Dhaubhadel

**Affiliations:** Agriculture and Agri-Food Canada, London Research and Development Centre, London, Ontario, Canada; University of Guelph, CANADA

## Abstract

In southern Ontario, Canada, the two-spotted spider mite (*Tetranychus urticae*) is an emerging pest of soybean (*Glycine max*) due to the increasing incidence of warmer, drier weather conditions. One key strategy to manage soybean pests is breeding resistant cultivars. Resistance to pathogens and herbivores in soybean has been associated with isoflavonoid phytoalexins, a group of specialized metabolites commonly associated with root, leaf and seed tissues. A survey of 18 Ontario soybean cultivars for spider mite resistance included evaluations of antibiosis and tolerance in relation to isoflavonoid and other metabolites detected in the leaves. Ten-day and 4-week trials beginning with early growth stage plants were used to compare survival, growth, fecundity as well as damage to leaves. Two-spotted spider mite (TSSM) counts were correlated with HPLC measurements of isoflavonoid concentration in the leaves and global metabolite profiling by high resolution LC-MS to identify other metabolites unique to the most resistant (R) and susceptible (S) cultivars. Within 10 days, no significant difference (P>0.05) in resistance to TSSM was determined between cultivars, but after 4 weeks, one cultivar, OAC Avatar, was revealed to have the lowest number of adult TSSMs and their eggs. Other cultivars showing partial resistance included OAC Wallace and OAC Lakeview, while Pagoda was the most tolerant to TSSM feeding. A low, positive correlation between isoflavonoid concentrations and TSSM counts and feeding damage indicated these compounds alone do not explain the range of resistance or tolerance observed. In contrast, other metabolite features were significantly different (P<0.05) in R versus S cultivars. In the presence of TSSM, the R cultivars had significantly greater (P<0.05) concentrations of the free amino acids Trp, Val, Thr, Glu, Asp and His relative to S cultivars. Furthermore, the R cultivar metabolites detected are viable targets for more in-depth analysis of their potential roles in TSSM defense.

## Introduction

Soybean (*Glycine max* L Merr) is an important field crop in southern Canada, and has been grown in Ontario for over 70 years. Most of the soybean acreage is glyphosate-tolerant (75%) used for oil and animal feed, while the remainder is non-GMO which is grown for food and organic production [1]. Soybean cultivars are selected by growers based on yield and the maturity group (MG) for that region. In southwestern Ontario, the MGs range from MG I to MG III, and the cultivars are selected by their performance in field trials which also consider some disease and pest resistance. Leaf-feeding soybean arthropod pests in Ontario include the soybean aphid (*Aphis glycines* Matsumura) (Hempitera: Aphididae), bean leaf beetle (*Certoma trifurcate* Forster) (Coleoptera: Chrysomelidae) and the two-spotted spider mite (*Tetranychus urticae* Koch) (Acari: Tetranychidae). The two-spotted spider mite (TSSM) feeds on individual plant cell contents on the underside of leaves through stylet-like mouthparts, causing yellowing, curling and bronzing of the leaves. Eventually, the infested leaf dries and falls off, affecting the soybean yield. TSSM damage is more severe in hot, dry weather, usually in mid-July in Ontario, Canada [1]. It was reported in early July 2020 that soybean fields in Ontario had high spider mite numbers and growers were applying chemical sprays [2]. No calculation of economic threshold for TSSM in Ontario soybean has been provided, but growers are encouraged to use a registered acaricide when the numbers per leaflet exceed 4 mites. Similarly, precise data on damage caused by mites in Ontario soybean is lacking, but in similar latitudes in the US Midwest there are records of damage caused by mites where it has been estimated that spider mite damage can reduce yields as much as 40–60% [3]. Studies in Brazil found that economic injury is apparent with 1 spider mite per cm^2^ leaf area even when there is little apparent damage or visible chlorosis [4]. The strategies for integrated pest management (IPM) in soybean involves monitoring and application of insecticides to reduce the pest pressure. Insecticides are applied to control high populations of aphids and mites, however the over-use of insecticides can cause mite populations to increase by reducing native enemies (ladybird beetles and predatory mites). Another IPM component is host plant resistance, defined as a heritable decrease in plant susceptibility to the pest [5]. Reducing the number of insecticide sprays or delaying applications until later in the growing season by slowing mite population growth is the goal of using more resistant soybean cultivars.

Research to identify aphid resistant soybean varieties has discovered several genes that confer one or more categories of resistance to this insect, including antibiosis (affects survival, growth and fecundity), antixenosis (affects behaviour such as oviposition) and tolerance (plant can withstand greater damage without economic loss) [5–8]. Aphid resistant lines are not commonly a target for breeding in non-GMO soybean and growers don’t tend to choose the resistant varieties (personal communication, Grain Farmers of Ontario). Similarly, even less emphasis has been given to TSSM resistance in local soybean cultivars. However, with the increasing spider mite damage in southern Ontario, developing TSSM resistant cultivars could become one of the breeding priorities in soybean.

Host plant resistance is generally cultivar-specific and defined by factors such as chemical and/or physical defenses. An important phytochemical defense in legumes are the isoflavonoid phytoalexins, that are induced upon disease infestation. Pathogens induce soybean leaf levels of isoflavonoid aglycones (daidzein, formononetin, isoformononetin, genistein, glycitein and glyceollins I-III), isoflavone glycosides (daidzin, genistin, ononin) and the malonylglycoside conjugates (malonyldaidzin and malonylgenistin) with varying concentrations and accumulation between cultivars [9]. Genistein, daidzein, glycitein, and their conjugates are found in high amounts in leaf and embryo tissues during late seed development stage while soybean seeds contain more of the malonyl glycosides, all of which act to modulate microbial interactions [10]. Isoflavonoids can also provide host plant resistance to insects, such as aphids and stink bugs [11,12]. Since both stink bugs and aphids are phloem feeding, they would encounter isoflavones as soluble and mobile glycosylated derivatives that are present in the soybean phloem [10]. Feeding behaviour of the pea aphid (*Acyrthosiphon pisum*) was prolonged by genistein, as demonstrated by the longer period of stylet probing and reduced salivation and passive ingestion [13]. A soybean genotype ‘Zhongdou 27’ from China with more than twice the amount of daidzein, 80% more genistein, 50% more glycitein and almost double the total isoflavones was more resistant to soybean aphid [12]. It was also observed that aphid attack led to increased levels of daidzein and genistein in the resistant but that response was not observed in the susceptible genotypes. Screening the amounts of phytoalexins in resistant cultivars is promoted as a way to identify insect-resistant germplasm for breeding programmes. For example, multivariate analysis indicated daidzein and genistein were the most important discriminators that reduced damage by the seed pod-feeding brown stink bug (*Euschistus heros*) in soybean [14].

Fewer investigations have focused on soybean resistance to herbivorous mites, such as TSSM, a generalist herbivore that feeds on over 1100 plant species including 150 crops [15]. In several soybean-growing regions, including the United States [16], Brazil [17,18] and Iran [19,20], interest in host resistance to mite species has led to discoveries of cultivar differences. In this study, we sought to determine which of the locally available cultivars in Ontario have the greatest TSSM resistance and to investigate whether tolerance or resistance is associated with isoflavonoids or other metabolites present in the leaf. The objectives of the research were: 1) to investigate genetic resistance to the TSSM in selected Ontario soybean cultivars and 2) to identify compounds associated with the resistance.

## Materials and methods

### Plants

Soybean (*Glycine max* [L.] Merr.) seeds were obtained from germplasm banks (Agriculture and Agri-Food Canada, London Research and Development Centre), and from Drs. I. Rajcan and M. Eskandari (University of Guelph, Guelph, Ontario). The seeds were planted in pots with Pro-Mix BX Mycorrhizae^TM^ soil (Rivière-du-Loup, QC, Canada) and grown in a growth chamber set at 23°C under light intensity 250 μmol photons m^-2^s^-1^ for 16:8 hours light:dark (L:D) cycle with 60–70% relative humidity (RH). Plants were fertilized with N:P:K 20:8:20 and watered with reversed osmosis (RO) water when required.

### Spider mites

A TSSM (*Tetranychus urticae*) colony was obtained from the London Research and Development Centre (Agriculture and Agri-Food Canada) and transferred from kidney bean and maintained on a low isoflavonoid soybean cultivar, Beer Friend. Separate mesh cages (50 cm^3^) held two soybean plants and were infested with TSSM. Soybean plants were replaced as required. The cages with plants and TSSM were kept in a growth chamber at 26 ± 2°C, 50 ± 5% RH and a 16:8 L:D photoperiod. A fresh 3–4 week old plant was added to each mesh cage every 1–2 weeks after removing the oldest plant.

### TSSM resistance assessment

#### Ten day trial–survival, growth and fecundity on a soybean leaf

*A 10 day period was chosen to assess the short-term response of TSSM over a range of leaf* isoflavonoids represented by selected soybean cultivars as it encompasses the life-cycle of TSSM from larva to reproductive adult. Soybean plants at the V3-V4 stage when third or fourth trifoliate leaves had developed were grown under the conditions described for use in a 10 day bioassay with TSSM to determine differences in nymph and larval growth and fecundity of adults that develop within one generation based on the methods developed for assessment of soybean aphid resistance [21]. Soybean cultivars representative of high, mid and low isoflavonoid leaf levels during the early growth stages (V1 to V3) were selected for testing. TSSM larval age was synchronized by collecting all larvae emerging from eggs that were deposited by females during a 24 h period after the transfer on to a fresh soybean leaf. To transfer TSSM larvae (< 1 day old) from the infested leaf to the soybean plant, 5 larvae were transferred with a single bristle brush on to a small leaf disk (cut from soybean cultivar Beer Friend leaf with a #3 dia cork borer). Lanolin was spread around the petiole to isolate mites to the leaf and a paper clip, wooden stake and pipe cleaners held back the adjacent leaves from the 2 isolated leaves as shown in S1 Fig. At the end of the 10^th^ day, the number of eggs, larvae + nymphs and adult TSSM on each leaf were counted to assess survival, growth and adult fecundity. Four 10 day trials were completed, with 4 separate cultivars/trial and three replicate plants/cultivar. For each cultivar, one plant without mites and one plant with the lanolin, paper clips, stake and pipe cleaners but no mites were used as controls for the TSSM and leaf isolation set-up. In total, 15 plants were held in a growth cabinet or environmental room with conditions set at 26 +2°C, 60 +5% RH, 250 μmol photons m^-2^s^-1^ light intensity, and 16:8 h L:D period.

After the TSSM and eggs were counted and removed, the 2^nd^ and 3^rd^ mid leaf and the mid leaf from the top 3 trifoliate leaves were sampled and placed in 3 separate 15 mL Falcon tubes, flash-frozen in liquid nitrogen and transferred to the -80°C freezer for storage for metabolite analysis.

#### Four week trial–sample counts and leaf damage assessment

A trial of 4 weeks duration was selected to cover a critical stage of soybean growth from early leaf stages (V1) to flowering and pre-seed pod development (R1-3) in order to compare TSSM population growth and feeding damage on plants with a range of isoflavonoid leaf levels. Soybean plants grown under the conditions described previously were selected at the V1 stage for use in a four-week trial with TSSM. After 4 weeks the level of infestation on the top leaves of the plants was determined by counting the eggs, larvae, nymphs and adult TSSM and measuring the feeding damage. Three greenhouse trials consisting of 4 cultivars with control and TSSM treatments were conducted for a 4-week period based on methods used in a previous study [21]. Each V1 plant at the beginning of the trial was enclosed in a white mesh cage (50 cm^3^) as shown in S2 Fig. Control plants for each cultivar received no mites at Week 0. For the TSSM treatment, plants were inoculated with five adult females on the first full mid leaf, then enclosed in the cage. All the plant cages with control and TSSM treatments were transferred to the green house with environmental condition maintained at 24 ±2°C, 50 ±20% RH, 250 μmol light, 16:8 h L:D. Each week (Weeks 1–3) cages were monitored for plant growth, damage, soil moisture and were watered as required. Plants and soil were sprayed lightly with a fertilizer solution (20-8-20, 0.5 g/L water) each week.

After 4 weeks (Week 4), the leaves were removed from the plant and photographed (top and bottom sides), and then scanned (top side only) with an Expression 11000XL photo scanner (Epson, Markham, ON, Canada). All leaves from each of the 3 trifoliate leaf levels were combined in 3 separate 15 mL Falcon tubes, flash frozen in liquid nitrogen and transferred for storage in the -80°C freezer. The TSSM infestation was visually rated for each plant by counting the numbers per leaf on the top 3 trifoliate leaves (top and bottom sides) and completing a damage assessment using percent leaf area damage. Antibiosis was assessed based on the average count of TSSM (mite and eggs) per trifoliate leaves for each cultivar. Tolerance was assessed using the percent leaf area damage by comparing the colour of control plant leaves to TSSM infested leaves (WinFOLIA Pro, Regent Instruments, Nepean, ON, Canada). For each cultivar, new assessment parameters were established in WinFOLIA for the colour of the healthy control leaves first, and then the colour of mite damaged areas on the infested leaves. The program analyzed the amount of healthy leaf colour on the mite-infested leaf which was then converted to percent leaf damage based on the difference.

### Isoflavonoid analyses

Leaf tissues were ground and metabolites were extracted in 1:1 (v/v) acetonitrile: water as described previously [10,22]. To convert malonyl- and acetyl-isoflavonoids conjugates to their corresponding glycosides, while leaving the majority of glycosides intact, a mild-hydrolysis of 100 μL leaf extracts was achieved by adding 50 μL 5% KOH solution, incubating at room temperature for 5 h, followed by neutralization with 50 μL 14% KH_2_PO_4_. HPLC analyses was performed following methods previously developed [22,23] using an Agilent 1200 series HPLC. A C18 column (Symmetry^®^, 4.6x250 mm) was used with a mobile-phase gradient of 10–35% acetonitrile in 0.1% acetic acid over 45 min at a flow rate of 1 mL min^-1^ and the detector set at 260 nm. The concentrations of aglycones (daidzein, glyceitin and genistein), and their glycosides (daidzin, glycitin and genistin) were identified and quantified by comparison of retention time and UV spectra to their corresponding standards. Standards of each isoflavonoid were purchased (Sigma-Aldrich).

### Metabolomic profiling

In order to identify chemical features associated with R or S cultivars in the presence or absence of TSSM, metabolomic profiling of the non-hydrolyzed extract was performed. One mL of ice cooled methanol water (4:1 *v/v*) was added to 50 mg of the leaf tissue and vortexed for 20s. The samples were than sonicated in a water bath filled with ice for 15 min and centrifuged at 11,000× *g* for 10 min at 4°C. Supernatant (700 μL) was removed and dried under nitrogen. The residue was reconstituted in 380 μL of methanol:water (1:1). The final solution was filtered with a 0.2 μm PTFE syringe filter into amber glass HPLC vials for analysis.

The samples were analyzed using a Thermo Scientific ™ Q-Exactive™ Orbitrap Mass Spectrometer, coupled to an Agilent 1290 HPLC. The following conditions were used for heated electrospray ionization (HESI): positive capillary voltage 3.9 kV; negative capillary voltage 3.5 kV; capillary temperature, 330°C; sheath gas, 32 arbitrary units; auxiliary gas, 10 units; probe heater temperature, 280°C and S-Lens RF level, 50%. Analytes were resolved by hydrophilic liquid interaction chromatography (HILIC) with a 350 μL min^-1^ flow rate. Samples (3 μL) were injected onto a Agilent HILIC-Z (2.1 × 100 mm, 2.7 μm; Agilent) column maintained at 35°C. Compounds were resolved with mobile phases of 20 mM ammonium formate in water (A) and 20 mM ammonium formate in 90% acetonitrile (B) operating with the following gradient: 0 min, 100% B; 0.5min, 100% B; 5.3 min, 80% B; 9.5 min, 30% B; 13.5 min, 30% B, 14.5 min 100% B and 15.5 min, 90% B. Samples were analyzed in both positive and negative ionization modes, by data-dependent acquisition. This consisted of a full MS scan at 70,000 resolution, automatic gain control (AGC) of 5×10^5^, maximum injection time (IT) of 256 ms and mass range 70 to 950 *m/z*. The top 3 ions were then analyzed by MS/MS at 17,500 resolution, 1×10^6^ AGC, 64 ms, 1.2 m/z isolation window and 28 normalized collision energy.

Thermo.raw files were converted to.mzml format using Proteowizard, with peak filter applied [24]. Features were detected using the XCMS package [25] with the centWave method [26] (ppm tolerance 1.0). The signal to noise threshold was set to 5, noise was set to 5×10^6^ and pre-filter was set to six scans with a minimum 5,000 intensity. Retention time correction was conducted using the obiwarp method [27]. Grouping of features was set to those present in at least 25% of all samples (retention time deviation 10 s; m/z width, 0.015). The ‘fillPeaks’ function was applied with default settings. Remaining zero values were input with two thirds the minimum value on a per mass basis. Compounds were identified by comparison of retention times and *m/z* to authentic standards or by accurate mass and comparison of fragmentation patterns to MS/MS databases. Amino acid concentrations in each sample were estimated by external calibration (TraceCert® Amino Acids Mix Solution, Millipore Sigma, St. Louis, MO) under the assumption that signal suppression/enhancement would be approximately equal across all soybean leaf samples. This data has been submitted to the NIH Common Fund’s National Metabolomics Data Repository (NMDR) website, the Metabolomics Workbench, https://www.metabolomicsworkbench.org.

### Statistical analyses

Two-way analysis of variance (ANOVA) was used to assess significant effects between the fixed factors cultivar and trifoliate leaf for the isoflavonoid concentration and the TSSM number and fecundity parameters. A Tukey’s test was applied to measure post hoc comparisons. Data was transformed when necessary and data that failed normality and homogeneity tests were analyzed using non-parametric tests: Kruskal-Wallis with comparisons performed using a Dunn’s test. The number of TSSM at the egg, or larval + nymph + adult stages, were correlated with the isoflavonoid concentration for each soybean cultivar at the growth stages associated with the sampling date using Pearson Correlation Log Rank analyses. Metabolite data from 2 R and 2 S cultivars was analyzed with two-way ANOVA using type-two error statements (R software, version 3.2.5, and the tool R studio) [28]. Principal Component Analysis (PCA) was conducted using the “FactoMineR” package with pareto scaling with the R software (version 3.2.5).

## Results

### Total isoflavonoid levels in soybean leaves

**Fig 1:**
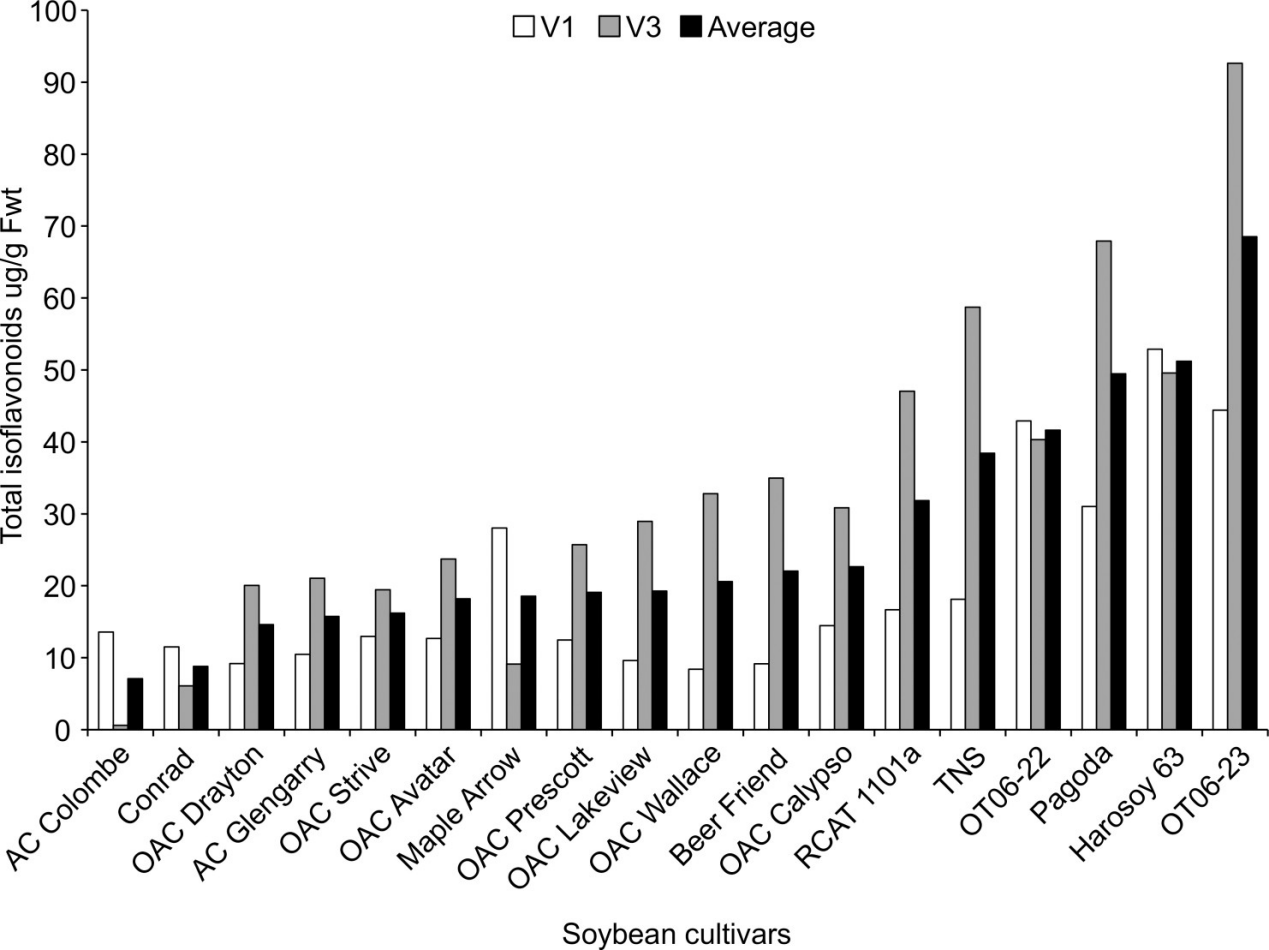
Total isoflavonoid concentration in the top trifoliate leaves of 18 soybean cultivars at the V1 and V3 growth stages and the mean concentration of both stages. The concentration of isoflavonoids in the top leaves was used to categorize the soybean cultivar as low, medium or high.

To measure the leaf isoflavonoid levels in Ontario grown soybean cultivars, leaf extracts from 18 different soybean cultivars including Beer Friend (a popular Japanese cultivar grown for Edamame, and used to maintain the TSSM colony in the lab) were analyzed at V1 and V3 stages for the presence of 3 isoflavone aglyones and their 3 corresponding glycosides (Fig 1). The analysis revealed that soybean leaf contains genistein and its glycoside genistin as the predominant isoflavonoids in leaf tissue (S3 Fig).

Based on the total leaf isoflavonoid content at the V1 and V3 stages (Fig 1), soybean cultivars were categorized into low, medium and high, and representative cultivars from each group were used for further TSSM trials. For example, the leaf isoflavonoid levels at the V3 stage, AC Colombe, Conrad and Maple Arrow were considered low; OAC Wallace, Beer Friend, OT06-22 and Harosoy 63 were moderately high; and RCAT 1101a, Pagoda and OT06-23 were the highest (Table 1). At the V1 stage, soybean cultivars OAC Wallace, OAC Lakeview, AC Glengarry, Conrad were considered low; OAC Avatar, OAC Strive, AC Colombe, Maple Arrow were considered as moderately high and Pagoda, OT06-22, OT06-23, Harosoy 63 were considered high (Table 2). The remaining 4 cultivars (OAC Drayton, OAC Prescott, OAC Calypso, TNS) were not used in the resistance evaluation as low, medium and high isoflavonoid cultivars were represented by the previous cultivars. This categorization of the soybean cultivars was maintained throughout the successive figures in order to keep the same order of presentation.

**Table 1:** Total isoflavonoid concentration (μg/g fresh weight) in control plants after the 10 day trial beginning at the V3 growth stage.

Cultivar	V3^1^	2^nd^	3^rd^	Top	Avg.^2^ (S.E.)
AC Colombe	low	1.4	2.8	5.9	3.4 (0.86)^a^
Conrad	low	20.0	7.1	7.9	11.7 (3.53)^abc^
Maple Arrow	low	0	26.6	0	8.9 (5.68)^ab^
OAC Wallace	mid^3^	9.4	10.1	10.9	10.1 (2.25)^abc^
Beer Friend	mid	12.2	10.7	10.7	11.2 (0.79)^abc^
RCAT 1101a	mid	1.3	35.1	32.5	19.8 (6.47)^bc^
Harosoy 63	mid	13.2	29.5	24.1	22. 3 (4.42)^c^
OT06-22	mid	5.8	20.9	39.4	22.0 (6.16)^c^
Pagoda	high	3.6	10.5	16.8	10.3 (2.63)^abc^
OT06-23	high	1.0	10.7	17.2	9.6 (3.01)^ab^

^1^Ratings at V3 stage were established with preliminary total isoflavonoid screening shown in Fig 1

^2^Ten day average isoflavonoid concentrations with the same lower case letters are not statistically different (Kruskal-Wallis, P>0.05)

^3^Mid refers to medium or moderately high isoflavonoid concentrations.

**Table 2:** Total isoflavonoid concentration (μg/g fresh weight) in control plants after the 4 week trial beginning at the V1 growth stage.

Cultivar	V1^1^	Top 1	Top 2	Top 3	Avg.^2^ (S.E.)
OAC Lakeview	low	9.1	5.9	7.5	7.5 (0.92)^a^
AC Glengarry	low	7.0	5.7	19.5	10.7 (4.40)^ab^
Conrad	low	11.2	15.8	21.0	16.0 (2.82)^abc^
OAC Avatar	low	31.7	23.4	23.8	26.3 (2.68)^bce^
OAC Strive	low	12.1	9.8	26.3	16.1 (5.15)^abc^
AC Colombe	low	10.0	11.0	12.2	11.1 (0.64)^ab^
OAC Wallace	low	44.1	28.7	58.5	43.8 (8.59)^de^
Maple Arrow	mid	37.8	34.6	41.1	37.9 (1.87)^cde^
Pagoda	mid	17.8	23.6	27.9	23.1 (2.93)^abcde^
Harosoy 63	high	50.3	48.3	56.3	51.6 (2.41)^e^
OT06-22	high	34.9	43.8	13.8	30.8 (8.91)^bcde^
OT06-23	high	40.1	15.4	14.2	23.3 (8.45)^abcde^

^1^Ratings at V1 stage were established with preliminary total isoflavonoid screening shown in Fig 1

^2^Four week average isoflavonoid concentrations with the same lower case letters are not statistically different (Kruskal-Wallis, P>0.05).

#### Ten day assessment of soybean cultivars response to TSSM

**Fig 2:**
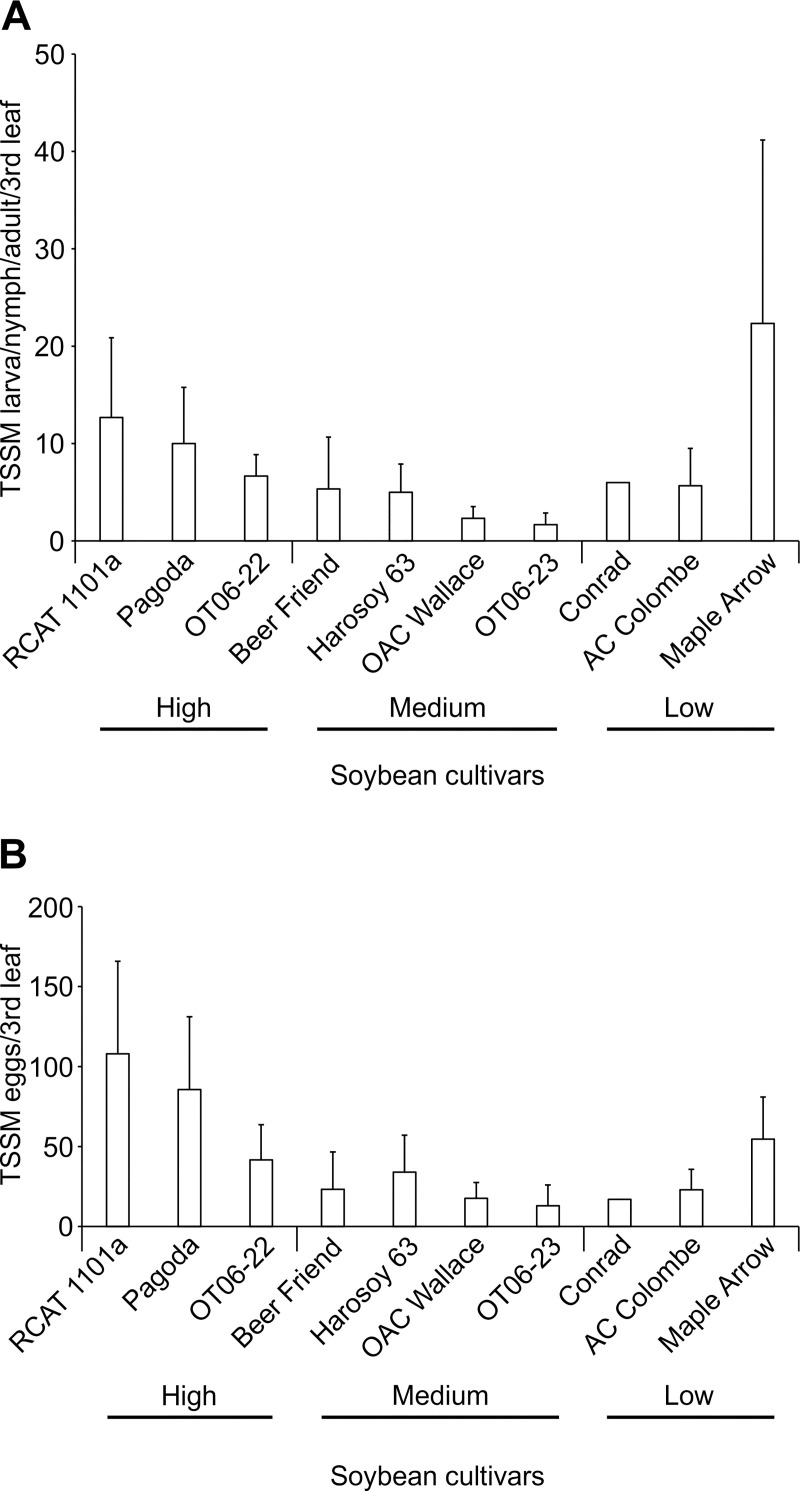
The performance of two-spotted spider mite on the 2^nd^ and 3^rd^ full leaf of 10 soybean cultivars after 10 days feeding. Data are the mean (± S.E.) TSSM larvae, nymph, adult and egg counts. No significant difference in TSSM larva, nymph and adult (Kruskal-Wallis, P>0.05) or TSSM egg (Kruskal-Wallis, P>0.05) numbers was revealed between the different cultivars tested beginning at the V3 stage.

Ten of the 18 soybean cultivars shown in Fig 1 were selected for the 10 day trials based on their differential leaf total isoflavonoid levels at the V3 stage. Ten days after placing 5 TSSM larvae on the 3^rd^ leaf, the TSSM larvae/nymphs/adults as well as the eggs were counted on the 3^rd^ leaves (Fig 2A and 2B). The cultivars Maple Arrow, RCAT 1101a and Pagoda had the highest average TSSM counts, ranging from 10.0 to 22.3 on the 3^rd^ leaf, but these numbers were not significantly greater (Kruskal-Wallis; d.f. = 9; chi-sq. = 5.1385; P = 0.8221) than observed on the cultivars with fewer TSSM present, OAC Wallace and OT06 23 (Fig 2A). The pattern of TSSM egg numbers on the 3^rd^ leaves of the 10 cultivars after 10 days was similar to the larva/nymph/adult TSSM numbers, and no significant difference in average egg counts was revealed between the cultivars (Kruskal-Wallis; d.f. = 9; chi-sq. = 6.0756; P = 0.7323) (Fig 2B).

**Fig 3:**
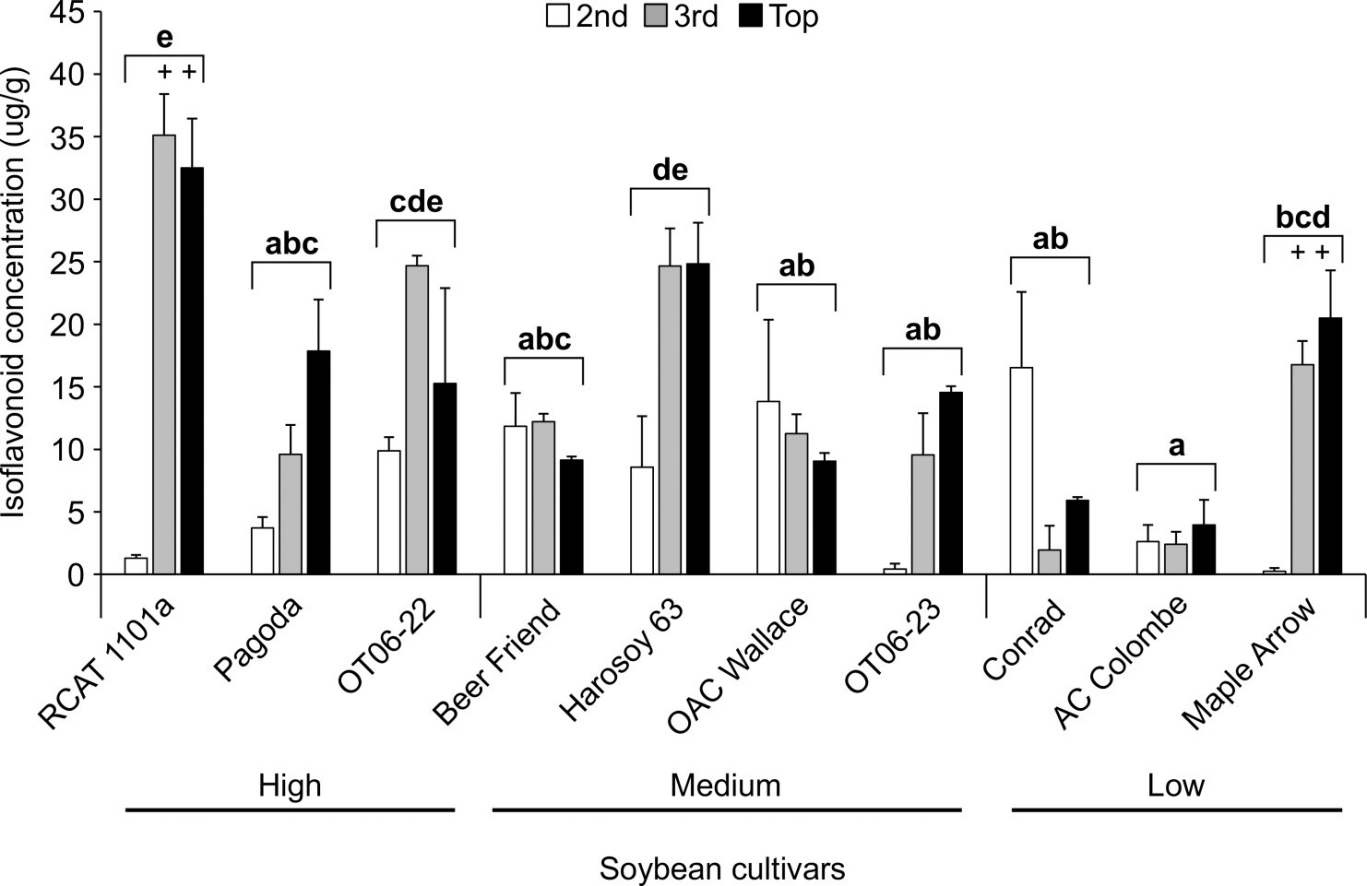
The effect of two-spotted spider mite feeding on isoflavonoid concentrations in the 2^nd^, 3^rd^ and top full leaves of 10 soybean cultivars after 10 days. Data are mean (± S.E.) isoflavonoid concentrations (μg/g). Bracketed bars for each cultivar with the same letters are not statistically different (2-way ANOVA, Tukey’s pairwise comparison, P>0.05). Analysis of the mid-leaflet of the trifoliate leaves indicated that there was no significant difference (P>0.05) in isoflavonoid concentrations between the 3 leaf levels, with the exception of 2 cultivars where the 2^nd^ full leaves had lower (++ = P<0.05) isoflavonoids than the 3^rd^ full and top leaflets.

At the end of the 10 day trial, analysis of total isoflavonoids in leaves of control soybean cultivars were conducted with 2^nd^, 3^rd^ and the top (Top) full leaflets. In the control plants, RCAT 1101a and Harosoy 63 had the highest isoflavonoid levels (Kruskal-Wallis; d.f. = 9; chi-sq. = 17.286; P = 0.0444), while AC Colombe had the lowest (Table 1). After 10 days, the TSSM-treated plants that had significantly higher isoflavonoids were RCAT 1101a, Harosoy 63 and OT06-22 (Two-way ANOVA; d.f. = 9,58; F = 11.957; P< 0.001) relative to OAC Wallace, Conrad and AC Colombe (Fig 3). The mean isoflavonoid contents for the 2^nd^ leaves were in general lower than 3^rd^ and top full leaves as shown by the significant interaction between cultivar and trifoliate leaf (Two-way ANOVA; d.f. = 19,58; F = 6.953; P< 0.0001). Statistical analysis established that total isoflavonoid concentrations in the 2^nd^ full leaves were significantly less than the 3^rd^ and top leaves (Top) for RCAT 1101a (Tukey’s test; P< 0.05) and significantly less than the Top leaves for Maple Arrow (Tukey’s test; P< 0.05). Based on these findings, only the TSSM counts after 10 days on the 3^rd^ full leaf were compared to the isoflavonoid concentration in those leaves. There was a low correlation between the 3^rd^ leaf isoflavonoid concentration and the number of TSSM larvae/adults (Pearson Log-Rank; r = 0.157; d.f. = 26; t = 0.8093; P = 0.4257) and eggs (Pearson Log-Rank; r = 0.18; d.f. = 26; t = 0.9314; P = 0.3602) for the 10 cultivars tested indicating that the isoflavonoid levels measured had a minimal effect on the survival or fecundity of TSSM.

#### Four week assessment of TSSM resistant and susceptible cultivars

**Fig 4:**
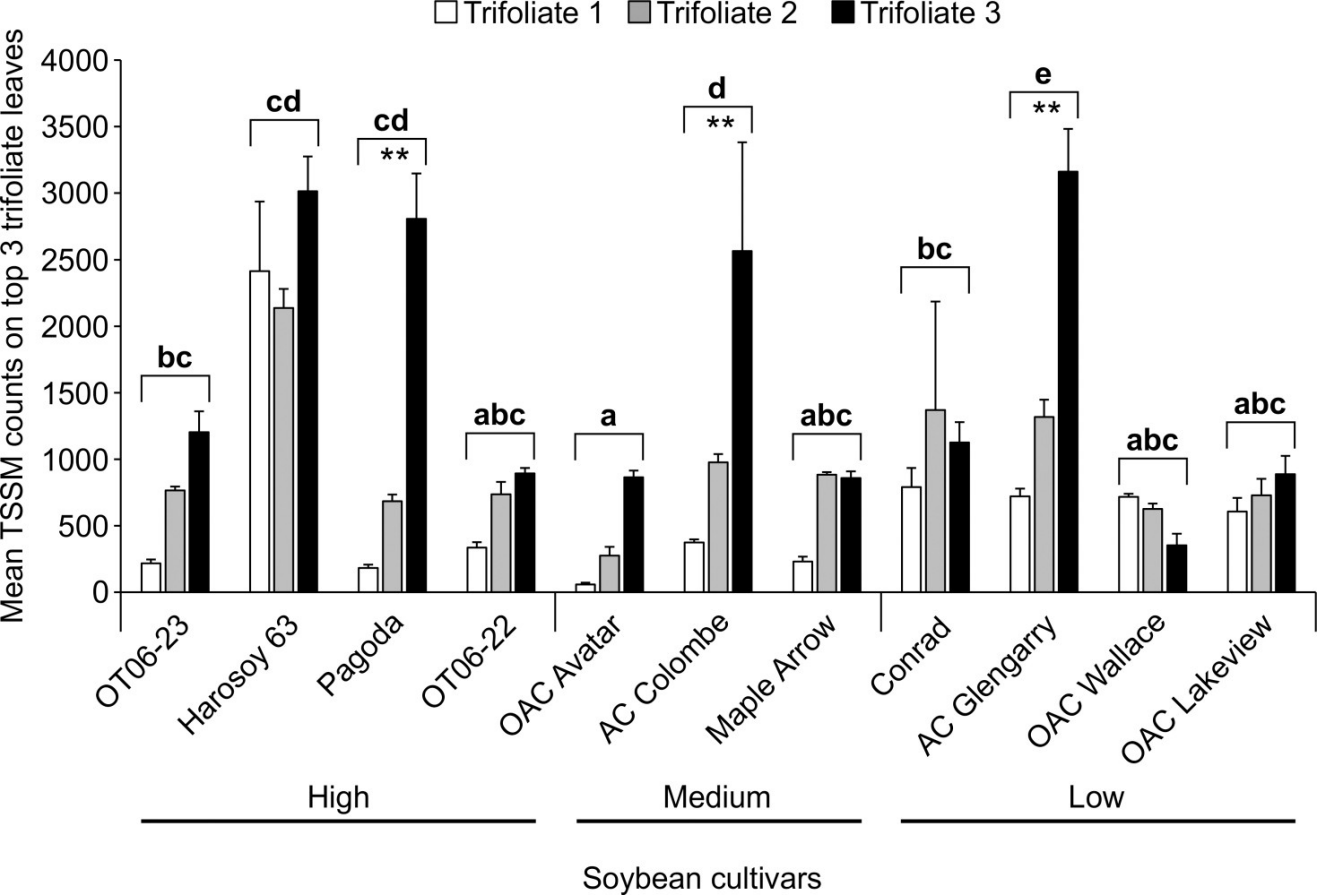
The performance of two-spotted spider mite on the top 3 trifoliate leaves of 11 soybean cultivars after 4 weeks. Data are mean (± S.E.) TSSM larvae, nymph, adult and egg counts. Bars with the same letters are not statistically different (2-way ANOVA, Tukey’s pairwise comparison, P>0.05). Analysis of the 3 youngest trifoliate leaves indicated that 3^rd^ full leaves generally had higher (** = P<0.05) TSSM counts than the 2^nd^ and top trifoliate for the most susceptible cultivars.

The resistance to TSSM based on average number of larval, nymph and adult mites and eggs on the 3 younger trifoliate leaves by the end of the 4-week greenhouse trial indicated that OAC Avatar was the most resistant cultivar (Two-Way ANOVA; d.f. = 10,66; F = 11.392; P< 0.0001), but 4 other cultivars had similar mite and egg counts: OAC Wallace, Pagoda, OAC Lakeview, OT06-22 and OT06-23 (P> 0.05) (Fig 4). Based on the number of TSSM and eggs on 2^nd^ trifoliate leaves, the remaining cultivars had on average 1.2 to 2.8-fold higher numbers of mites and eggs on the younger leaves than the more resistant cultivar. There was a significant interaction between cultivar and trifoliate leaf (Two Way ANOVA; d.f. = 20,66; F = 6.219; P< 0.0001), and 3 of the 4 least resistant cultivars (AC Glengarry, Pagoda, AC Colombe) had significantly greater differences in the numbers of mites and eggs between the top, second and third trifoliate leaves relative to the 3 most resistant cultivars (OAC Avatar, Maple Arrow, OAC Wallace).

**Fig 5:**
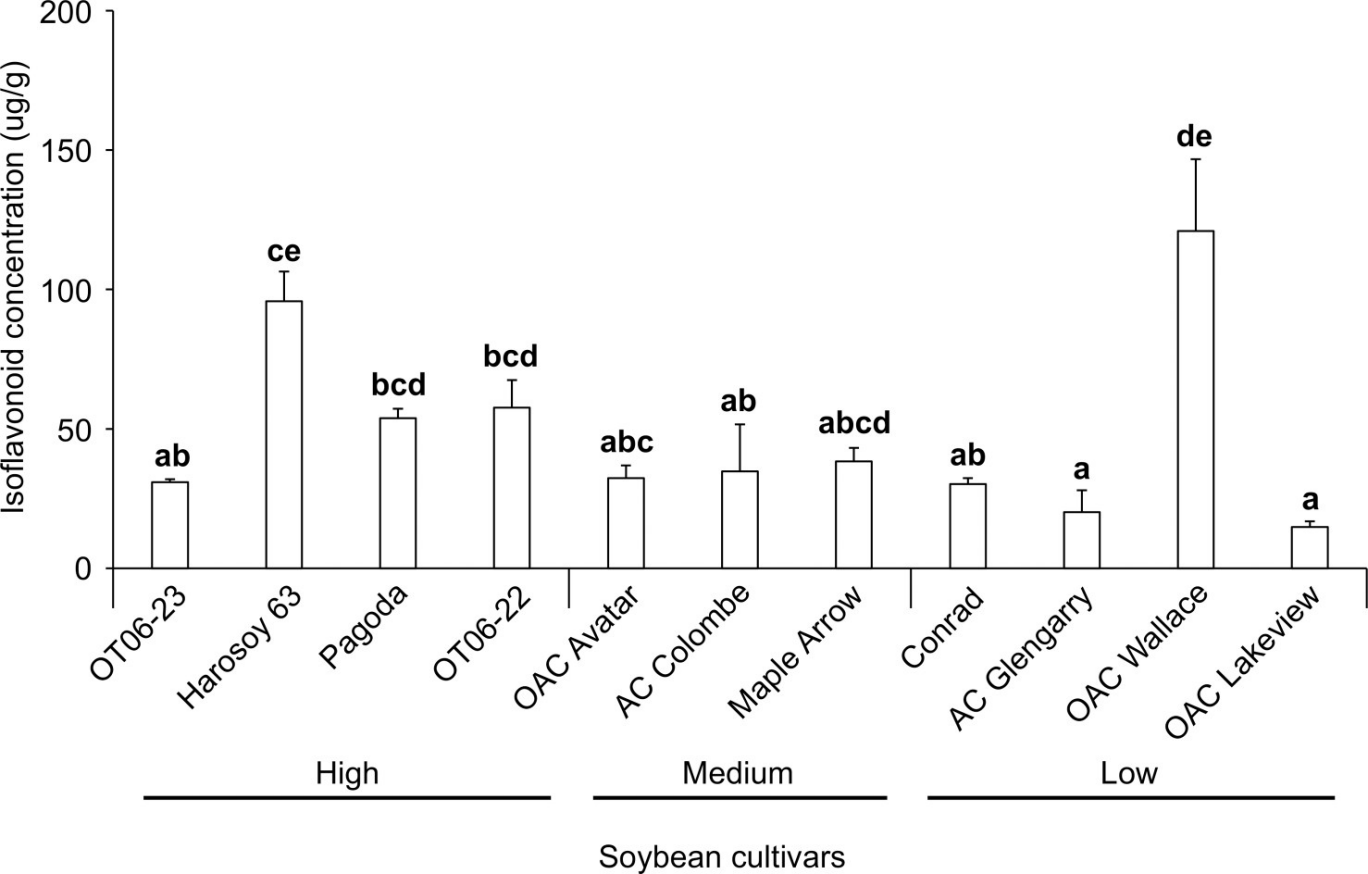
The effect of two-spotted spider mite feeding on total isoflavonoid concentrations in the top 3 trifoliate leaves of 11 soybean cultivars after 4 weeks. Data are mean (± S.E.) total isoflavonoid concentrations (μg/g). Bars with the same letters are not statistically different (Kruskal-Wallis, P>0.05).

After 4 weeks, soybean plants without mites had isoflavonoid concentrations that averaged from 7.5 to 51.6 μg/g in the top three trifoliate leaves (Table 2). Only the three cultivars with the highest levels, Harosoy 63, OAC Wallace and Maple Arrow, were significantly different from the three with the lowest concentration, AC Colombe, AC Glengarry and OAC Lakeview (Kruskal-Wallis; d.f. = 11; chi-sq. = 27.474; P = 0.0039). After four weeks of TSSM feeding, the 2 cultivars with the highest concentration of isoflavonoids (95.8 to 120.9 μg/g) in the top trifoliate leaves were OAC Wallace and Harosoy 63 (Fig 5). The leaves of AC Glengarry and OAC Lakeview contained levels (14.8 to 20.2 μg/g) that were significantly lower than other cultivars included in the study (Kruskal-Wallis; d.f. = 11; chi-sq. = 29.113; P = 0.0022).

The relationship between isoflavonoid concentration in the top 3 sets of trifoliate leaves (Fig 5) and the TSSM larvae/nymph/adult and egg counts (Fig 4) on those leaves showed a low correlation for the 1^st^ (Pearson Log-Rank; r = 0.397; d.f. = 9; t = 1.297; P = 0.227), 2^nd^ trifoliate leaves (Pearson Log-Rank; r = 0.351; d.f. = 9; t = 1.123; P = 0.291) and 3^rd^ trifoliate leaves (Pearson Log-Rank; r = -0.077; d.f. = 9; t = -0.232; P = 0.822).

### Isoflavonoid leaf levels and tolerance to TSSM

#### Four week trial—TSSM leaf damage

**Fig 6:**
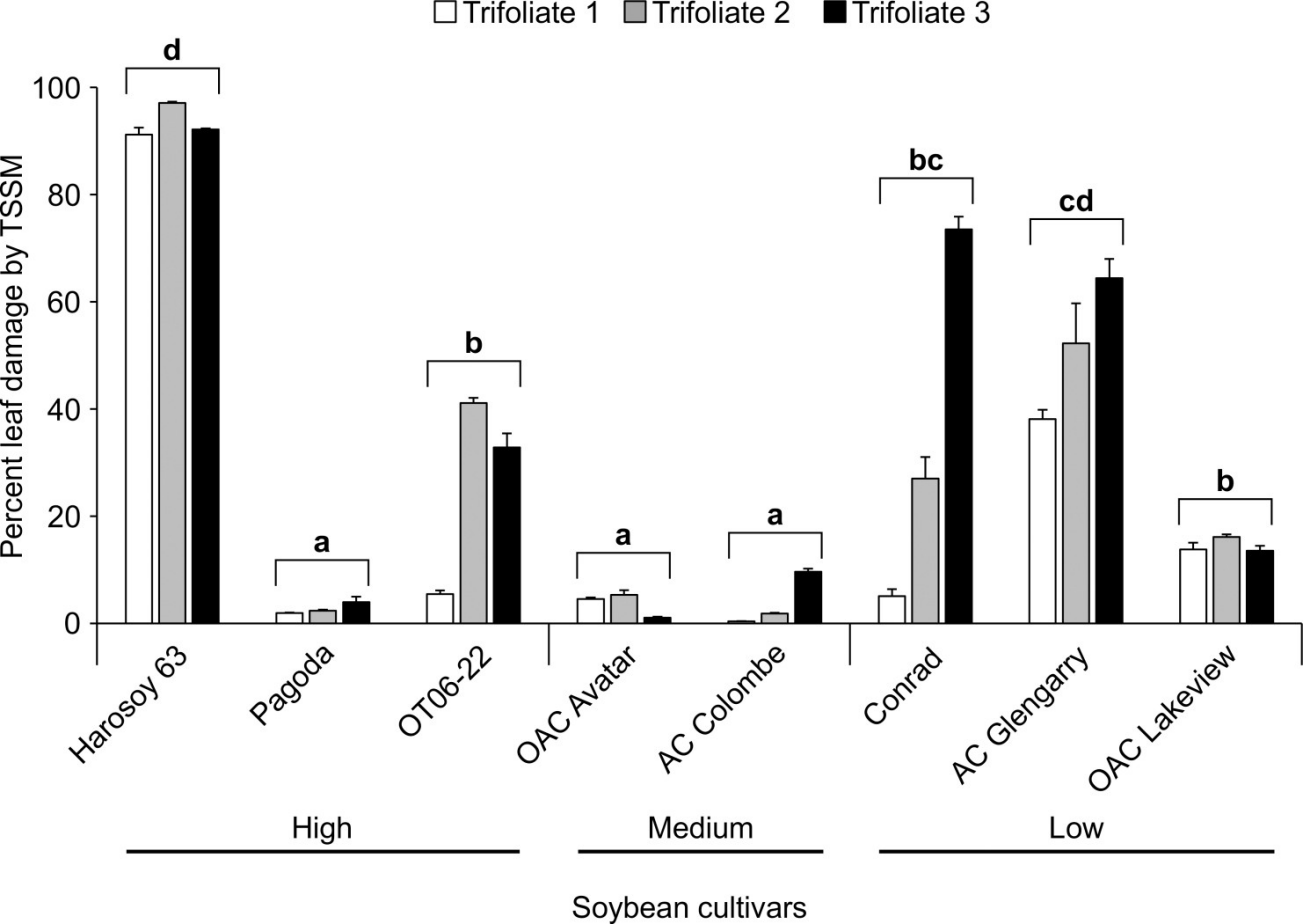
Leaf damage caused by two-spotted spider mite feeding to the top 3 trifoliate leaves of 8 soybean cultivars after 4 weeks. Data are mean (± S.E.) percent leaf damage. Bracketed bars with the same lower case letter are not statistically different (Kruskal-Wallis, Dunn’s test, P>0.05).

Damage to leaves of soybean by TSSM was assessed on the top 3 trifoliate leaves of 8 cultivars which represented a range of low to high isoflavonoids measured at the end of the 4 week trial (Fig 6). Percent damage to the leaves was ascertained using scanned images of the upper leaf surface of mite infested plants relative to leaves from control plants. The amount of feeding damage caused by TSSM indicated Harosoy 63 was the least tolerant (95% feeding damage) compared to AC Glengarry and Conrad (>35% leaf damage), while OT06-22 was moderately tolerant (26.5%) to damage relative to 3 of the remaining 4 cultivars (Kruskal-Wallis; d.f. = 7; chi-sq. = 55.758; P<0.0001). The most tolerant cultivars to TSSM feeding were Pagoda, OAC Avatar and AC Colombe with less than 5% damaged leaf surface (Dunn’s test; P<0.01858).

Damage on the TSSM infested plants showed a moderate correlation with isoflavonoid concentration), but it was not significant (Pearson Log-Rank; r = 0.589; d.f. = 6; t = 1.783; P = 0.125). Of the 6 isoflavonoid standards that were used in the HPLC analyses of the leaf extracts, only genestin was measured consistently in the different cultivars. Generally, there was a low correlation between resistance (antibiosis and tolerance) and genistin as a measure of total isoflavonoids, indicating these compounds do not fully explain the range of resistance observed to TSSM.

### Metabolomic analyses

Based on the results of the 4 week TSSM and egg counts (Fig 4), as well as leaf damage (Fig 6), a subset of cultivars was selected for a comprehensive metabolomic profiling (Table 3). The Harosoy 63 and Conrad were designated as TSSM susceptible (S) cultivars as both were revealed to have significantly higher leaf damage after 4 weeks relative to OAC Avatar and Pagoda, but Pagoda had higher TSSM number than OAC Avatar, the more TSSM-resistant (R) cultivar. The leaf extracts prepared from the 4-week greenhouse trials were subjected to HILIC LC high resolution mass spectrometry to measure differences in the metabolite profiles of S and R cultivars in the presence or absence of TSSM.

**Table 3:** Relative spider mite resistance ratings for 4 Ontario grown soybean cultivars.

Soybean cv.	Mite resistance	Comments
Harosoy 63	Most Susceptible	High TSSM counts and feeding damage
Conrad	Partial Resistance	Fewer TSSM counts but still have high feeding damage
Pagoda	Partial Resistance	More tolerant to TSSM feeding damage than Harosoy 63 and Conrad
OAC Avatar	Most Resistant	Lowest TSSM counts

**Fig 7:**
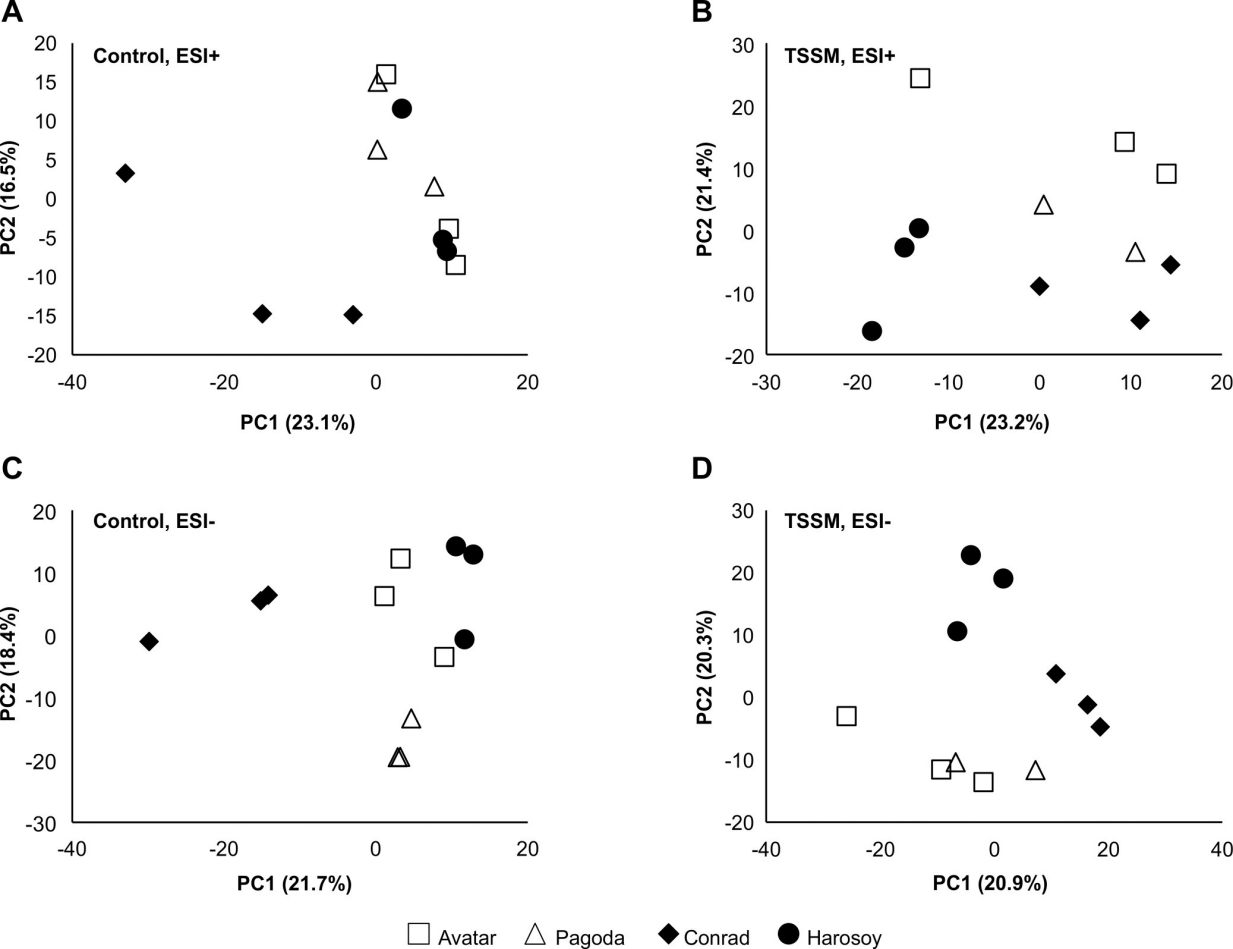
**PCA plots derived from XCMS extracted features of resistant (white) and susceptible cultivars (black).** (A) cultivars without mites, ESI^+^; (B) cultivars with mites, ESI^+^; (C) cultivars without mites, ESI^-^ and (D) cultivars with mites, ESI^-^. Each symbol represents one of 3 biological replicates.

A non-targeted analysis performed with XCMS identified a total of 637 molecular features in positive ionization mode. The samples were plotted using principle components analyses (PCA) which revealed there was overlap between the TSSM R cultivars, OAC Avatar and Pagoda, and the S cultivars, Harosoy 63 and Conrad, under control conditions (Fig 7A), and some clustering between R and S cultivars was observed under mite infestation conditions (Fig 7B) for the electrospray ionization, positive capillary voltage (ESI^+^).

With the negative ionization (ESI^-^), we observed no evident clustering between the R and S cultivars under control conditions (Fig 7C), however under TSSM treatments the R and S did form unique groups (Fig 7D). Overall, this indicated that there were no comprehensive chemical differences in the metabolite profiles of S and R cultivars under control conditions. Therefore, individual chemical features that were significantly different between R and S cultivars under TSSM conditions were examined.

Following the application of Benjamini-Hochberg multiple hypothesis testing correction, there were no molecular features that were significantly different (P > 0.05) between R and S cultivars in the control treatment. Conversely, in the TSSM treated samples, 12 features were significantly different (P<0.05), all being increased in the R cultivars. The most prominent included a peptide containing molecule of formula C_36_H_44_N_6_O_7_ whose identity is not known (S4A Fig), nicotinate ribonucleoside (S4B Fig) and a dipeptide, putatively identified methyl-lysyl-valine (S4C Fig). Of these 3 compounds, the unknown peptide had greater intensity and there was a significant effect of cultivar (Two-way ANOVA; d.f. = 3,15; F = 16.8201; P<0.0001) in TSSM-infested leaves of R relative to S cultivars (Table 4). Other metabolites that were significantly altered were primary amino acids (except cysteine) including, tyramine, cadaverine and ornithine, which were targeted for quantification (Table 5). Within the 2 R soybean cultivars, in only 1 (Pagoda) was there a significant increase in amino acid (Trp) concentration due to mite feeding (Two-Way ANOVA; d.f. = 1,15; F = 20.4713; P = 0.0004). With the S cultivars, Harosoy 63 and Conrad, there was a significant interaction between treatment and cultivar (Two-Way ANOVA; d.f. = 3,15; F = 32.047; P<0.0001) for Tyramine concentrations in mite infested leaves compared to the controls. Furthermore, there was a significant interaction between treatment and cultivar (Two-Way ANOVA; d.f. = 3,15; F = 4.4815; P = 0.015) for Ser where the concentration was significantly greater (Tukey’s test; P = 0.0122) in TSSM-infested leaves compared to un-infested leaves in Conrad. There were 4 amino acids where levels were higher (Tukey’s test; P<0.0352) in control leaves for R versus S cultivars, Leu, Ala, Gly and Tyramine. In 3 of these cases OAC Avatar was the R cultivar with the higher amino acid concentrations. On mite infested leaves, there were 6 cases where amino acids were greater (Tukey’s test; P<0.0474) for the R compared to the S cultivars, Val, Thr, Glu, Asp, Ser and His. In contrast, Tyramine levels were significantly greater (Tukey’s test; P<0.0224) in one S cultivar, Harosoy 63, versus the two R cultivars on the mite infested leaves.

**Table 4:** The effect of two-spotted spider mite feeding on peptides and nucleosides in leaves of 2 resistant and 2 susceptible soybean cultivars after 4 weeks.

	Resistant/tolerant cultivars								Susceptible cultivars							
	OAC Avatar				Pagoda				Harosoy 63				Conrad			
Amino acids	Control (E+07)^1^		TSSM (E+07)		Control (E+07)		TSSM (E+07)		Control (E+07)		TSSM (E+07)		Control (E+07)		TSSM (E+07)	
	Avg	(SE)	Avg	(SE)	Avg	(SE)	Avg	(SE)	Avg	(SE)	Avg	(SE)	Avg	(SE)	Avg	(SE)
^**2**^ **Pep.**	7.24	(1.79) ^bc^	11.6	(1.1) ^c^	9.08	(2.74) ^bc^	11.2	(1.96) ^c^	6.05	(0.74) ^abc^	3.25	(0.81) ^ab^	0.002	(0.0) ^a^	2.63	(0.45) ^ab^
^**3**^ **NR.**	8.83	(2.70) ^ab^	18.7	(3.11) ^b^	11.2	(3.15) ^ab^	18.7	(3.95) ^b^	10.4	(2.51) ^ab^	3.48	(0.56) ^a^	7.06	(1.11) ^ab^	8.52	(2.19) ^ab^
^**4**^ **ML.**	1.56	(0.63) ^a^	1.89	(0.13) ^a^	1.61	(0.28) ^a^	2.14	(0.28) ^a^	0.88	(0.07) ^a^	0.78	(0.02) ^a^	0.24	(0.07) ^a^	0.37	(0.03) ^a^

^1^Average peak area (x E+07) ± standard error (SE)

^2^Unkown peptide

^3^nicotinate D-ribonucleoside

^4^methyl_lysyl-valine. Peptides and nucleoside in a row with the same letters are not statistically different (2-way ANOVA, P>0.05).

**Table 5:** The effect of two-spotted spider mite feeding on amino acid concentrations in leaves of 2 resistant and 2 susceptible soybean cultivars after 4 weeks.

	Resistant/tolerant cultivars								Susceptible cultivars							
	OAC Avatar				Pagoda				Harosoy 63				Conrad			
Amino acids	Control		TSSM		Control		TSSM		Control		TSSM		Control		TSSM	
	Avg^1^	(SE)	Avg	(SE)	Avg	(SE)	Avg	(SE)	Avg	(SE)	Avg	(SE)	Avg	(SE)	Avg	(SE)
**Phe**	62	(42) ^a^	54	(38) ^a^	21	(3) ^a^	73	(4) ^a^	25	(9) ^a^	39	(33) ^a^	10	(7) ^a^	51	(18) ^a^
**Trp**	58	(25) ^ab^	106	(62) ^b^	13	(1) ^a^	127	(36) ^b^	53	(8) ^ab^	80	(42) ^ab^	10	(4) ^a^	68	(19) ^ab^
**Ile**	20	(12) ^a^	13	(7) ^a^	6	(2) ^a^	10	(3) ^a^	11	(5) ^a^	10	(8) ^a^	8	(6) ^a^	10	(3) ^a^
**Leu**	32	(15) ^b^	20	(12) ^ab^	10	(2) ^ab^	13	(6) ^ab^	20	^ab^	15	(10) ^ab^	3	^a^	11	^ab^
**Met**	0.3	(0.4) ^a^	0.14	(0.04) ^a^	0.73	(0) ^a^	1	(2) ^a^	1	(0.29) ^a^	0.2	(0.1) ^a^	1	(1) ^a^	0.20	(0.2) ^a^
**Tyr**	17	(14) ^a^	25	(25) ^a^	6	(5) ^a^	24	(11) ^a^	12	(5) ^a^	7	(4) ^a^	7	(1) ^a^	12	(3) ^a^
**Val**	228	(62) ^ab^	325	(34) ^b^	157	(63) ^a^	169	(52) ^ab^	245	(83) ^ab^	150	(46) ^a^	127	(49) ^a^	135	(62) ^a^
**Pro**	53	(24) ^a^	40	(20) ^a^	22	(8) ^a^	34	(13) ^a^	96	(25) ^a^	74	(103) ^a^	11	(2) ^a^	16	(3) ^a^
**Ala**	7	(2) ^a^	7	(3) ^a^	21	(7) ^b^	12	(3) ^ab^	6	(1) ^a^	5	(2) ^a^	4	(1) ^a^	7	(1) ^a^
**Thr**	36	(6) ^ab^	50	(10) ^ab^	51	(23) ^ab^	76	(28) ^b^	31	(13) ^ab^	24	(20) ^a^	16	(5) ^a^	31	(10) ^ab^
**Gly**	5	(1) ^b^	3	(0.2) ^ab^	3	(1) ^ab^	5	(1) ^b^	2	^a^	3	^ab^	2	^a^	4	(1) ^ab^
**Gln**	29	(2) ^ab^	29	(3) ^ab^	21	(4) ^ab^	65	(17) ^b^	14	(5) ^a^	52	(36) ^ab^	9	(1) ^a^	34	(15) ^ab^
**Ser**	46	(7) ^abc^	58	(9) ^bc^	46	(7) ^abc^	88	(46) ^c^	41	(7) ^abc^	33	(19) ^ab^	11	(4) ^a^	65	(11) ^bc^
**Asn**	11	(1) ^a^	11	^a^	32	(28) ^a^	33	(20) ^a^	9	(3) ^a^	14	(13) ^a^	6	^a^	9	^a^
**Glu**	640	(71) ^ab^	516	(181) ^ab^	656	(130) ^ab^	791	(151) ^b^	374	(145) ^ab^	262	(109) ^a^	294	(245) ^a^	341	(115) ^ab^
**Asp**	253	(42) ^ab^	318	(82) ^ab^	313	(137) ^ab^	648	(371) ^b^	186	(30) ^a^	203	(100) ^a^	110	(89) ^a^	242	(71) ^a^
**His**	15	^abc^	21	(7) ^c^	10	^abc^	19	(3) ^bc^	6	^ab^	18	(8) ^bc^	4	^a^	8	(3) ^ab^
**Lys**	9	(2) ^ab^	10	(4) ^ab^	11	(3) ^ab^	20	(3) ^b^	6	^a^	10	(5) ^ab^	14	(8) ^ab^	9	(1)^ab^
**Arg**	11	(5) ^a^	8	(4) ^a^	8	^a^	15	(6) ^a^	9	^a^	10	(3) ^a^	11	(3) ^a^	10	^a^
^**2**^ **Cadav.**	0.4	(0.3) ^a^	0.44	(0.1) ^a^	0.21	(0.0) ^a^	0.31	(0.04) ^a^	0.29	(0.1) ^a^	0.25	(0.1) ^a^	0.49	(0.5) ^a^	0.20	(0.0) ^a^
^**3**^ **Ornith.**	0.3	(0.1) ^ab^	0.34	(0.1) ^ab^	0.29	(0.1) ^ab^	0.81	(0.2) ^ab^	0.29	(0.1) ^ab^	0.87	(0.5) ^b^	0.10	(0.2) ^a^	0.50	(0.3) ^ab^
^**4**^ **Tyram.**	1.0	(0.4) ^bc^	1.2	(0.2) ^bc^	0.66	(0.4) ^ab^	0.67	(0.2) ^abc^	1.0	(0.3) ^bc^	3.8	(0.2) ^d^	0.15	(0.1) ^a^	1.4	(0.2) ^c^

^1^Mean concentrations (ng/g) ± standard error (SE) have not been corrected for extraction recovery or SSE%

^2^Cadaverine

^3^Ornithine

^4^Tyramine. Amino acids per row with the same letters are not statistically different (2-way ANOVA, P>0.05).

## Discussion

The present study identified antibiosis resistance and tolerance within MG II Ontario soybean cultivars to the generalist herbivore, TSSM. Experiments conducted over a 4 week period during the pre-seed development stage of soybean development (Fig 4) identified cultivars where TSSM abundance was significantly less on the top trifoliate leaves of one cultivar, OAC Avatar, compared to many of the other cultivars tested. A second cultivar, Pagoda, had measurable tolerance, even though it experienced high TSSM abundance on the top leaves (Fig 6). In contrast, the shorter duration trial, 10 days in length, found no statistical differences in TSSM larvae/nymph/adult abundance (Fig 2A), or egg abundance (Fig 2B), although there was a similar trend observed in abundance across the 10 cultivars. Chromatographic analyses of defense compounds in the leaves (Figs 3 and 5) indicated that selected isoflavonoids were not correlated with antibiosis resistance or tolerance to leaf damage. Mass spectrometry was used to compare leaf extracts prepared from TSSM resistant (R) and susceptible (S) cultivars with and without TSSM feeding. The analysis revealed metabolite profiles differed between TSSM R and S cultivars (Fig 7D), possibly based on concentrations of free amino acids and diamines (Table 5) and an unknown peptide (Table 4), indicative of greater feeding damage in the leaves of S cultivars. The molecular formula of the peptide-like compound was determined to be C_36_H_44_N_6_O_7_ and immonium ions for proline (*m/z* 72.0815), leucine/isoleucine (*m/z* 86.0969) and tryptophan (*m/z* 159.0916), however the full identity could not be ascertained at this point. These differences also represent the soybean response to the TSSM feeding not observed in leaves unaffected by TSSM.

Previously, studies of chemical changes due to TSSM feeding on soybean leaves established that resistant cultivars had initially higher levels of lipoxygenase (LOX) and peroxidase (POX) activity, in part a response to leaf damage and presence of oxygen radicals, but also an effective defense to TSSM [16,29]. Other investigations of soybean host resistance to TSSM examined solely the effect of genotype on life history parameters under laboratory [19,20] and field conditions [17]. In all cases, the differences in life history were attributed to plant quality, including nutrients, morphology and allelochemical profiles. The present research advances knowledge on soybean host-plant defenses further by identifying classes of foliar compounds that are different in cultivars with lower TSSM abundance and leaf damage.

Screening of herbivore resistance in existing soybean varieties for antibiosis, antixenosis and tolerance has been documented more often in soybean aphids [5,6,8,21,30] than with TSSM [16,19,20]. The association of aphid resistance in soybean with phytochemical defenses is not as common as it is with 5 resistance genes (*Rag 1*- *Rag 5*) that are correlated with negative effects to biology and behaviour [31]. A recent review of the potential mechanisms of *Rag* gene action summarized that the induction of these genes is related to secondary metabolite production, but the metabolites (isoflavonoids, phenolics, and others) levels were not correlated with changes in aphid feeding behavior in order to advance knowledge on host plant resistance [32]. Currently, there is no known genetic association with resistance to TSSM so the cultivars screened in the present research were compared by leaf chemistry. Some cultivars were found to have increasing isoflavonoid concentrations in new growth trifoliate leaves over the early growth period (OAC Avatar and RCAT 1101a), while others declined during this period (Harosoy 63 and OT06-22), remained the same (Conrad), or initially increased but then declined (OAC Wallace, OAC Lakeview, AC Glengarry, OAC Strive, Pagoda and OT06-23) (Fig 1 and Table 1). No pattern emerges from this information that suggests that either higher isoflavonoid concentrations in early or later stages is predictive of TSSM resistance, with the exception of OAC Wallace that had one of the lower TSSM abundance and the highest isoflavonoid concentrations in top leaves after 4 weeks. As TSSM resistance had not been evaluated in soybean over a time frame such as 10 days, it was thought a shorter period might provide similar predictive results as the 4 week trial. However, 10 days was not found to be a suitable length of time to separate the resistant from susceptible cultivars, in part due to the higher variability in mite survival and fecundity. Soybean accessions tested with a similar sized herbivore (soybean aphid) found that it did not move far from the inoculated trifoliate during that period, but it was recommended instead to use aphid counts on the whole plants in the late vegetative or early reproductive to determine the resistant accessions [21]. This demonstrates the importance of choosing the appropriate plant growth stage and assay type to be able to correctly determine the host plant resistance for a particular herbivore. Trials to determine aphid resistance between genotypes found that when experiments began at the V3 stage greater resistance was observed than those that began at V1 [6], and differences in resistance to aphids of several biotypes were noted between whole plants and detached leaves from those plants [30]. Differences between plant stage and length of feeding time were not as evident for spider mite resistance, in part because no significant difference was revealed between all cultivars after 10 days (Fig 2A and 2B) from V3 to approximately V6, whereas there was a range of TSSM resistance observed over 4 weeks (Fig 4) encompassing V1 to R3. Under field conditions, spider mites begin infesting soybean from the V5 stage, and the density increases greatly up to R5 for most cultivars, with the growth rate peaking in the reproductive stages (between R2 and R5) [17]. Based on the field measurements of growth, it is better to use plants at later growth stages to determine plant resistance to TSSM.

The induction of isoflavonoids due to TSSM feeding was observed in the 4 week trials where the average concentration doubled in the top 3 trifoliate leaves of TSSM infested plants (Fig 5) relative to the control plants (Table 2). The induction was not consistent across all cultivars, and the increase was not observed with the same TSSM resistant cultivars. In a recent study [33], there were significant increases in the leaf levels of the isoflavones daidzein, genistein, and formononetin caused by aphids over a 21 day period. Transcriptome analysis found there were no differences between aphid-infested versus un-infested resistant plants with the Rag1 gene after 21-days, indicating the resistant soybean lines had higher constitutive defenses not present in the susceptible lines. Soybean herbivory by other insects did induce isoflavonoids in resistant cultivars, for example, seed levels of genestein and daidzein, by the stink bug (*Euschistus heros*) [14]; leaf levels of the flavone and isoflavone aglycones 4’,7-dihyroxyflavone, daidzein, and formononetin, and also the isoflavone glucoside daidzin by *Spodoptera litura* (Lepidoptera: Noctuidae) feeding [9]; and changes in leaf phenolic compounds caused by *Spodoptera frugiperda* (J.E. Smith) feeding [34]. In contrast, changes in the metabolite profile were greater for TSSM susceptible cultivars, Conrad and Harosoy 63, compared to a TSSM resistant one, OAC Avatar, pre- and post-feeding by spider mites [35]. In either case, future research to compare the effects of soybean induced defenses between species from different feeding guilds would be valuable. The effect of induced flavonoids may be greater for lepidopteran species due in part to the difference in feeding behaviour between caterpillars and piercing-sucking hemipterans and acari more so because of the greater uptake of those compounds.

The wild soybean (*G*. *soja* Siebold and Zucc.) has been a source of aphid resistant accessions with different virulence biotypes for breeding resistance. Resistance in *G*. *soja* has been correlated with the daidzein content in the leaves thought to contribute to growth inhibitory effects with the Oriental leafworm (*Spodoptera litura*) [36]. In the present study, daidzein was not often detected in the cultivars using HPLC and was not one of the compounds found to differ significantly between R and S cultivars. Different isoflavonoids were also found to contribute to herbivore defense in wild relatives of other legumes, including chickpea (*Cicer arietinum* Maackiain and Judaicin), where anti-feedant activity was noted against the cotton bollworm (*Helicoverpa armigera*) [23].

Tolerance to TSSM was observed with some of the R cultivars tested, for example Pagoda, where less feeding damage was observed when the numbers of mites were high enough to cause significantly greater feeding damage relative to other cultivars. Differences in leaf damage may be due to characteristics of antibiosis, antixenosis or both for each cultivar. Antibiosis could be the reason for slower growth of a population on a resistant cultivar than on a more susceptible cultivar thus reducing the amount of damage [6]. This appeared to be the case for OAC Avatar where fewer TSSM were present relative to Harosoy 63 and the associated leaf damage was significantly less. Antixenosis could be more likely the cause when the numbers of mites per leaf were not statistically different yet leaf damage varied between cultivars. For example, OAC Lakeview and OT06-22 had similar numbers of TSSM to AC Colombe and Pagoda, but had significantly higher damage. However, separating antibiosis and antixenosis factors to test for tolerance may require a comparison at the genetic level. A new finding from our work was the association between certain free amino acids and TSSM resistance in soybean (Table 5), not documented previously for spider mites and soybean. TSSM, both those adapted and non-adapted to tomato defenses, were observed to induce free amino acids under drought conditions, including the essential amino acids, Val, Ile, Leu, Tyr, Phe, His, Lys and Arg and a non-essential amino acid Ser [37]. Similarly, tomato plants under stress from both drought and tomato red spider mite (*T*. *evansi*) infestation were found to have significantly induced levels of essential amino acids, Val, Ile, Leu, Tyr, His and nonessential Glutamic acid [38]. The drought conditions were thought to be mainly responsible for the higher essential amino acids levels, along with more available free sugars, which created better nutritional conditions to enhance growth and fecundity for both *T*. *urticae* and *T*. *evansi*. Another study that measured increased foliar amino acid levels caused by TSSM feeding was completed on strawberry *Fragaria* spp. [39]. In that case, the ratio of total amino acids:phenolics decreased over the 2 week feeding period, the mites appeared to adapt to the deficiency of amino acids and an excess of phenolic compounds.

Previous studies with soybean aphid found line-specific amino acid levels that were constitutive (prior to aphid feeding) or induced responses because of *A*. *glycines* feeding that altered amino acid levels between R and S plants [40]. The R line (*Rag1* gene) was thought to have a constitutively lower level of certain amino acids, whereas the S lines had higher concentrations of Aba, Asn, Gln, Glu, His, Pro and Ser. The effect of this difference to aphid development could be related to nutritional quality, thus explaining why the S line was more suitable for the aphids, or in contrast, why the *Rag1* line was considered resistant. Peach aphid (*Myzus persicae* Sulzer) leaf density was also associated with increasing total free amino acid content in pepper (*Capsicum annuum* L.) leaves, including aromatic amino acids (Phe, Tyr and Trp); the branched-chain amino acids (Val, Ile, and Leu); and amino acids from other groups (Arg, Lys, Met, Thr, Ala, Asn, and His) [41]. In contrast, metabolomic analyses of soybean R and S cultivars indicated certain amino acids (Tyr, Leu, Ile, Met, Val, Lys, and Glu) were higher in the R strain prior to foxglove aphid (*Aulacorthum solani* Kaltenbach) feeding, followed by increases in the levels of other metabolites, including flavonoids and alkaloids, over several hours of feeding [42]. In 15 soybean varieties, there was a positive correlation with Asn, Trp, Ala, Phe, and Ser, and a negative correlation with Leu and Thr for another piercing-sucking herbivore, the bean plataspid stink bug (*Megacopta cribraria* F.) [43]. Insects in other feeding groups also show a preference for soybeans with higher quantities of free essential amino acids [43,44]. The beet armyworm (*Spodoptera exigua* Hubner) was thought to respond to the greater diversity and abundance of free essential amino acids in a preferred host plant, pigweed (*Amaranthus hybridus* L.), compared to the less preferred cotton (*Gossypium hirsutum* L.), as it provided a better diet for the developing larvae [44]. Pigweed had Leu and 54% of the essential amino acids, whereas cotton had only 30% and no Leu. In seeds and seedlings of sorghum (*Sorghum bicolor* L. Moench), Ala, Pro, and Cys were significantly and negatively associated with 93–96% of the antibiosis and resistance to the spotted stem borer (*Chilo partellus* Swinhoe) [45]. When the amino acids were measured in *C*. *partellus* larvae that fed on susceptible and resistant sorghum genotypes, Ala, Cys, Gly and Pro were significantly and negatively correlated with antibiosis, with Gly having the strongest association with stem borer resistance. In the present study, Ala and Gly were significantly higher in R soybean cultivars relative to the S cultivars under control conditions, whereas Pro levels were no different and Cys was not measured. TSSM resistance that was observed after induction of defenses is associated with greater concentrations of Ser, Val, Thr, Glu, Asp and His, indicating a greater overlap with the foxglove aphid responses than those of other hemipteran species on soybean (Table 6).

**Table 6:** Amino acids associated with herbivore nutrition, feeding response or plant resistance.

Plant	Herbivore	Amino acids associated with			Reference
		Nutrition	Feeding response	Resistance	
Soybean	Two-spotted spider mite		^2^Tyramine	^1^Ala, Gly, Leu, Tyramine ^2^Asp, Glu, His, Ser, Thr, Val	Present study
Soybean	Fox-glove aphid			^1^Tyramine ^2^Glu, Ile, Leu, Lys, Met, Val, Tyr	32
Soybean	Soybean aphid	^1^Aba, Asn, Gln, Glu, His, Pro, Ser			30
Soybean	Bean plastapid stink bug	^1^Ala, Asn, Phe, Ser, Trp		^1^Leu, Thr	33
Tomato	Two-spotted spider mite	^2^Arg, His, Ile, Leu, Lys, Phe, Ser, Tyr, Val			37
Tomato	Tomato red spider mite	^2^Glu, His, Ile, Leu, Tyr, Val			38
Pepper	Peach aphid		^2^Ala, Arg, Asn, His, Ile, Leu, Lys, Met, Phe, Thr, Trp, Tyr, Val		31
Pigweed	Beet armyworm	^1^Leu			34
Sorghum	Spotted stem borer			^3^Ala, Cys, Pro ^4^Ala, Cys, Gly, Pro	35

^1^Consitutive levels

^2^induced levels

^3^in plant

^4^in larva.

Our findings do not explain how the presence of higher concentrations of certain free amino acids might affect TSSM growth and fecundity negatively, but induction of one non-essential amino acid, glutamic acid, in resistant soybean cultivars was measured under non-drought conditions, suggesting that TSSM feeding was more likely responsible. This information can provide a better prediction of which soybean cultivars are TSSM-resistant, or less preferred, and is an intriguing line of research to follow up.

Other compounds detected by mass spectrometry that were not previously associated with soybean-TSSM interactions were a nucleoside, nicotinate D-ribonucleoside, and a dipeptide, methyl-lysyl-valine (Table 4). However, neither were determined to differ in concentration between the R and S cultivars, with or without TSSM feeding. Other related nucleosides derived from bacteria, bagougeramine A and aspiculamycin, both with cytosine as a nucleobase and a glucopyranosyl sugar moiety, have acaricidal activity against TSSM [46]. The unidentified peptide (C_36_H_44_N_6_O_7_) in our study was present in greater concentrations in the R cultivars. This previously unknown peptide should be examined in greater detail as a novel TSSM defense in soybean.

As there was a low correlation between leaf isoflavonoids and the resistance to mites among the cultivars examined at 4 weeks post-infestation, it is likely that other phytochemicals including the free amino acids identified in R cultivars, were responsible for the range of reduced feeding and lower reproduction observed. Differences between soybean aphid, the soybean specialist, and TSSM, the generalist herbivore, include the type of leaf tissues fed on, phloem versus cellular, respectively. TSSM feed on over 1100 plant species, including 150 crops, due in part to its ability to evade most of the host defenses of a potential host [15]. Spider mites as generalist herbivores can adapt to many different plant defenses including constitutive and inducible metabolites and proteins. TSSM have the ability to digest, metabolize and transport many classes of xenobiotics from plant toxins to acaricides [15]. Another adaptive strategy is suppression of defenses by effectors in the TSSM saliva [47–49]. Adaptation by TSSM to a few of the same soybean cultivars from the present study observed that over 10 generations TSSM fecundity increased on a more resistant cultivar in part due to increased metabolism through enhanced cytochrome P450 activity [35].

The selection of currently available soybean aphid resistant cultivars can assist growers to minimize yield losses and reduce the amount of insecticide applied during the season. However, soybean cultivars resistant to aphids are not necessarily resistant to TSSM, an important point that soybean growers in Ontario and other regions should be aware. Future research should evaluate the promising resistant cultivars in the field and other cultivars identified as resistant to TSSM under laboratory and greenhouse conditions. The difference in metabolites between susceptible and resistant cultivars can be applied both as biomarkers to predict herbivore resistant cultivars and for use in breeding programs. The resistant cultivars may also provide valuable short-term assistance in order to reduce crop damage and yield loss as well as decreasing the number of pesticide applications and the associated costs.

## Supporting information

S1 Fig Isolated leaf on soybean plant to inhibit spider mite migration. Lanolin on the petiole (A) and paper clips and cotton pipe cleaners to separate the leaf from others leaves (B).
(TIF)

S2 Fig Soybean plant (OAC Wallace cv.), V1 stage, at the start of the 4 week greenhouse trial. (TIF)

S3 Fig Chromatographs of 6 isoflavonoid standards (A) and a typical methanol extract leaf sample (B).
(TIF)

S4 Fig MS/MS spectra of key features detected in higher abundance in TSSM resistant soybean cultivars. Peptide-like compound of formula C_36_H_44_N_6_O_7._ Immonium ions and neutral mass loss suggest the presence of Trp, Leu, Pro and Gly (A); Putatively identified as ribosyl-nicotinate based on accurate mass and matching MS/MS spectra found in METLIN (B); Feature shown in (C) has a molecular formula of C_12_H_25_N_3_O_3_ matching Lysyl-Leu/Ile, however key immonium ions at 72.08147 *m/z* and 84.08126 *m/z* suggest the presence of Val and Lys and not Leu/Ile.
(TIF)
