# Supplementary figures and images for: Soybean (Glycine max L Merr) host-plant defenses and resistance to the two-spotted spider mite (Tetranychus urticae Koch)
Source: PLoS One. 2021 Oct 7;16(10):e0258198. doi: 10.1371/journal.pone.0258198 (PMC8496822; doi:10.1371/journal.pone.0258198)

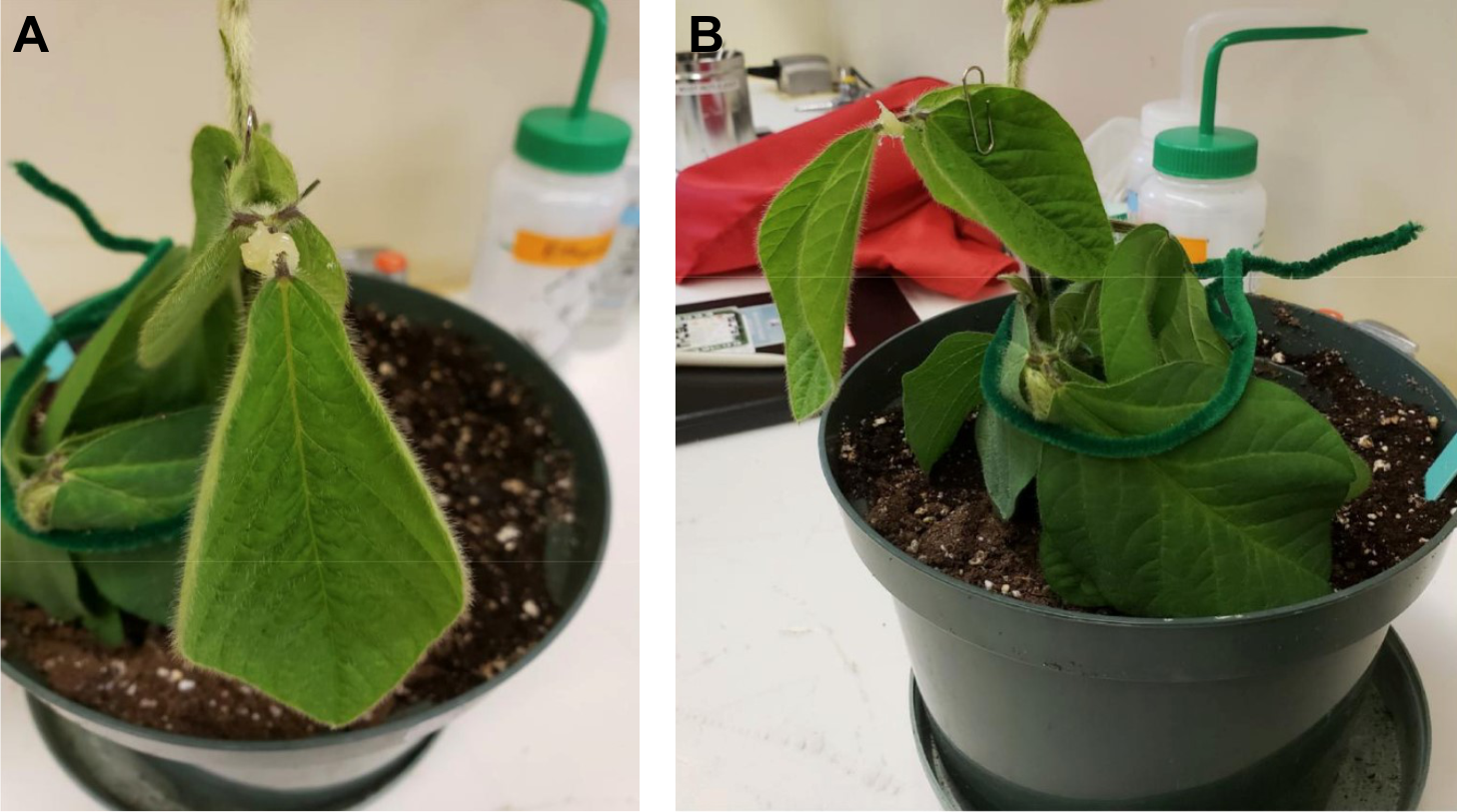

Supplement: S1 Fig — Lanolin on the petiole (A) and paper clips and cotton pipe cleaners to separate the leaf from others leaves (B). (TIF) [file pone.0258198.s001.tif]

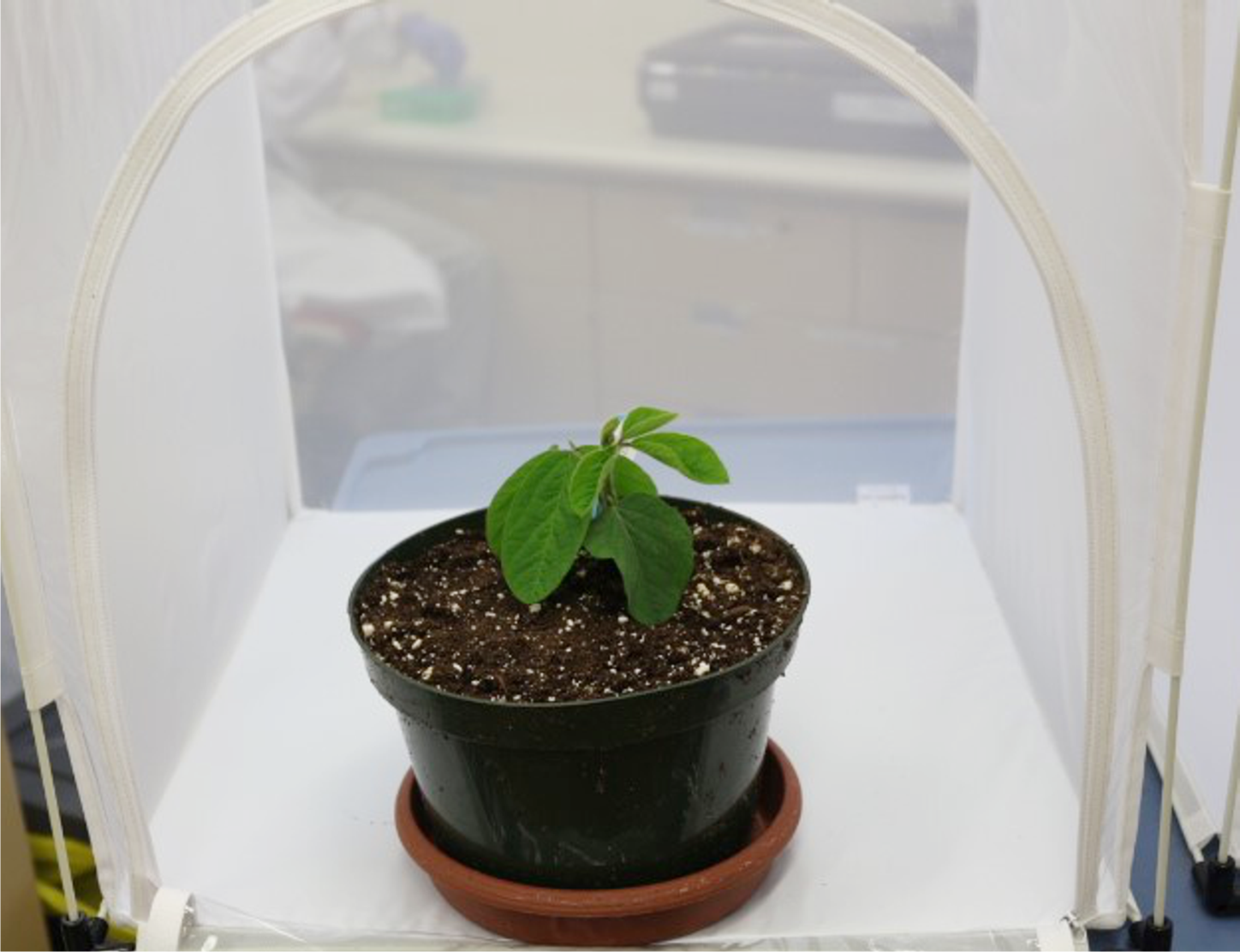

Supplement: S2 Fig — (TIF) [file pone.0258198.s002.tif]

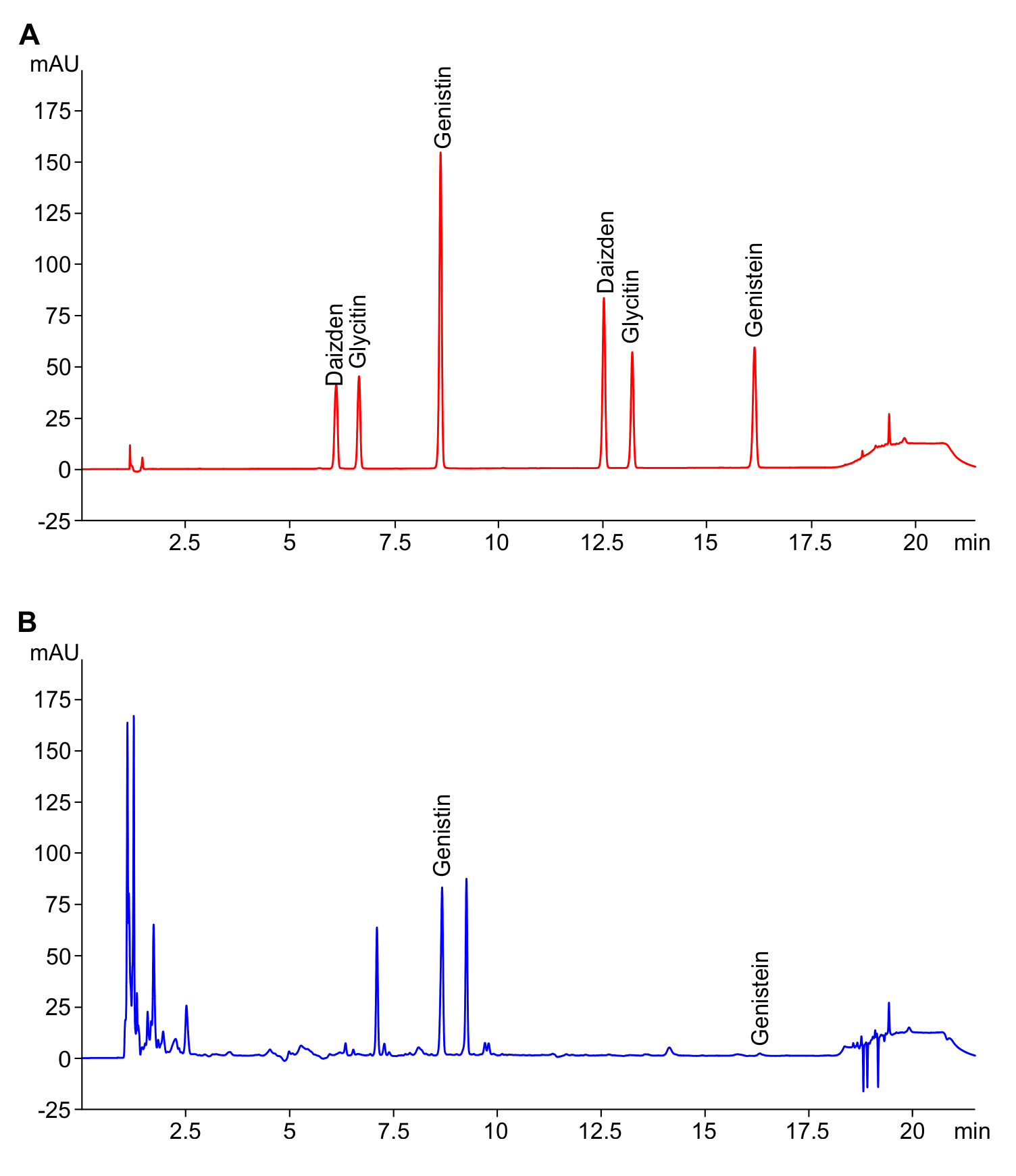

Supplement: S3 Fig — Chromatographs of 6 isoflavonoid standards (A) and a typical methanol extract leaf sample (B). (TIF) [file pone.0258198.s003.tif]

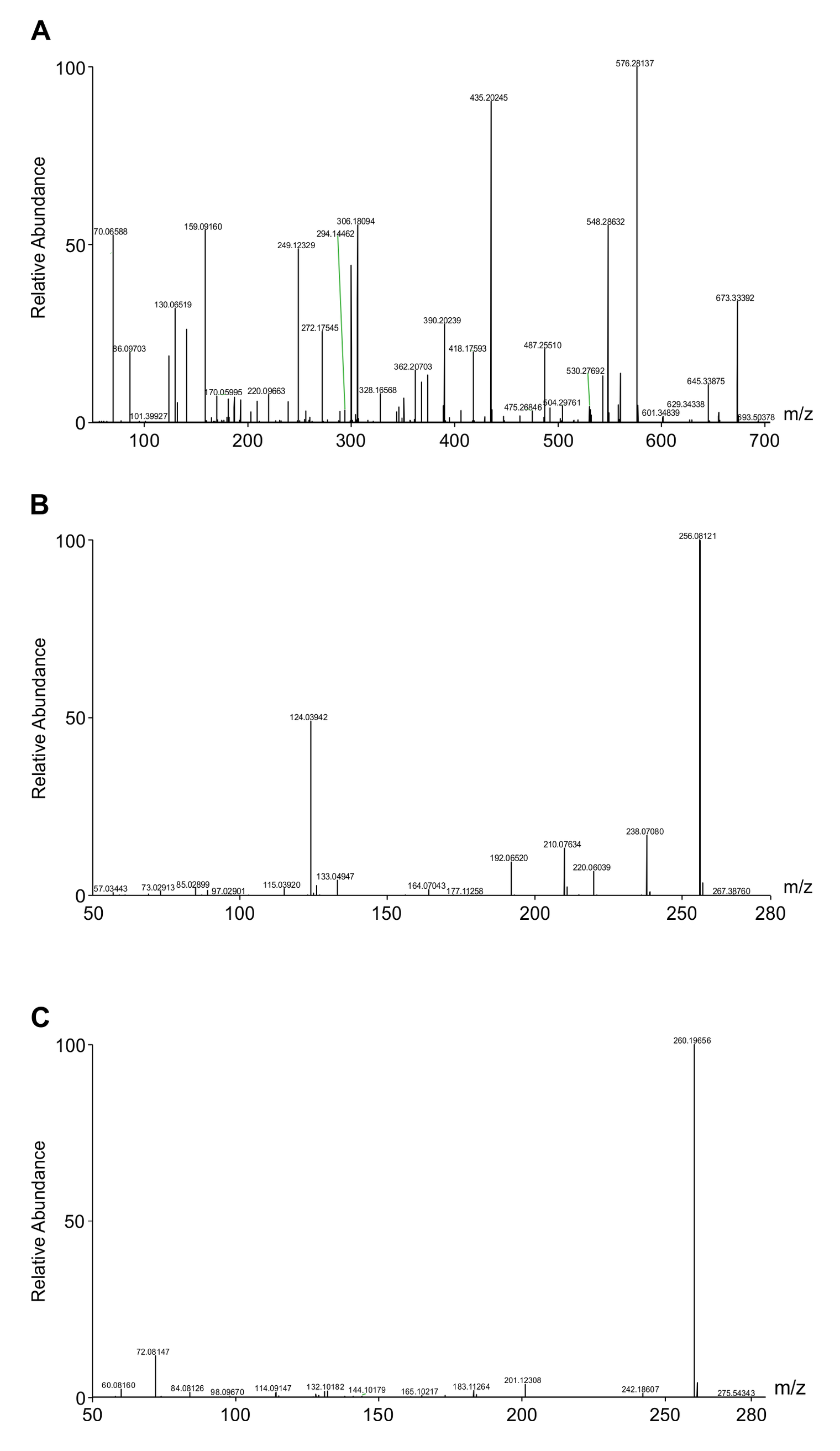

Supplement: S4 Fig — Peptide-like compound of formula C36H44N6O7. Immonium ions and neutral mass loss suggest the presence of Trp, Leu, Pro and Gly (A); Putatively identified as ribosyl-nicotinate based on accurate mass and matching MS/MS spectra found in METLIN (B); Feature shown in (C) has a molecular formula of C12H25N3O3 matching Lysyl-Leu/Ile, however key immonium ions at 72.08147 m/z and 84.08126 m/z suggest the presence of Val and Lys and not Leu/Ile. (TIF) [file pone.0258198.s004.tif]
